# Experimental and Theoretical Investigation of the Reaction of NH_2_ with NO at Very Low Temperatures

**DOI:** 10.1021/acs.jpca.3c03652

**Published:** 2023-08-17

**Authors:** Kevin M. Douglas, Daniel Lucas, Catherine Walsh, Mark A. Blitz, Dwayne E. Heard

**Affiliations:** † School of Chemistry, University of Leeds, Leeds LS2 9JT, U.K.; ‡ School of Physics and Astronomy, University of Leeds, Leeds LS2 9JT, U.K.; § National Centre for Atmospheric Science (NCAS), University of Leeds, Leeds LS2 9JT, U.K.

## Abstract

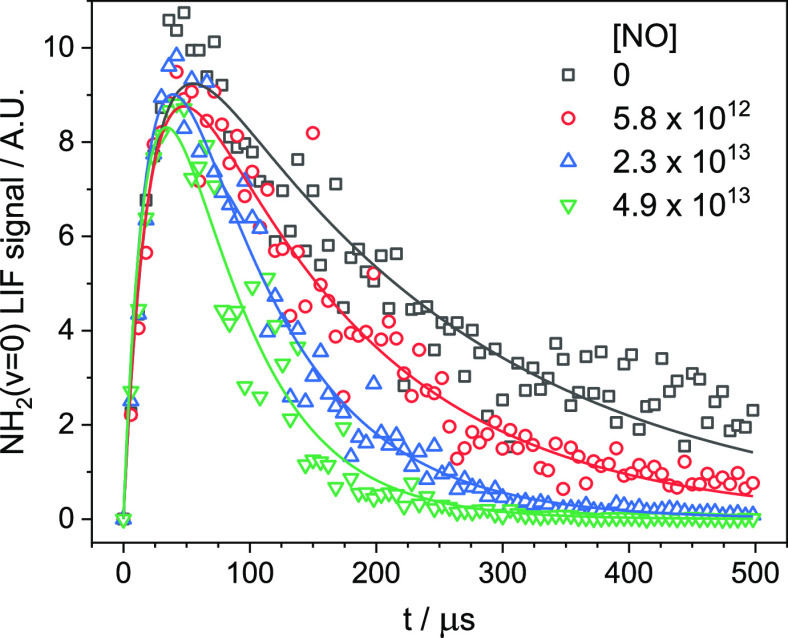

The first experimental study of the low-temperature kinetics of the gas-phase reaction between NH_2_ and NO has been performed. A pulsed laser photolysis-laser-induced fluorescence technique was used to create and monitor the temporal decay of NH_2_ in the presence of NO. Measurements were carried out over the temperature range of 24–106 K, with the low temperatures achieved using a pulsed Laval nozzle expansion. The negative temperature dependence of the reaction rate coefficient observed at higher temperatures in the literature continues at these lower temperatures, with the rate coefficient reaching 3.5 × 10^–10^ cm^3^ molecule^–1^ s^–1^ at *T* = 26 K. Ab initio calculations of the potential energy surface were combined with rate theory calculations using the MESMER software package in order to calculate and predict rate coefficients and branching ratios over a wide range of temperatures, which are largely consistent with experimentally determined literature values. These theoretical calculations indicate that at the low temperatures investigated for this reaction, only one product channel producing N_2_ + H_2_O is important. The rate coefficients determined in this study were used in a gas-phase astrochemical model. Models were run over a range of physical conditions appropriate for cold to warm molecular clouds (10 to 30 K; 10^4^ to 10^6^ cm^–3^), resulting in only minor changes (<1%) to the abundances of NH_2_ and NO at steady state. Hence, despite the observed increase in the rate at low temperatures, this mechanism is not a dominant loss mechanism for either NH_2_ or NO under dark cloud conditions.

## 1 Introduction

The reaction between NH_2_ and NO, *k*_1_, plays an important role in the thermal DeNO_*x*_ process,^1–5^ in which NO_*x*_ is thermally removed by NH_3_, and as such has been studied extensively both experimentally^6–25^ and theoretically.^10,24–36^ The reaction has three main product channels

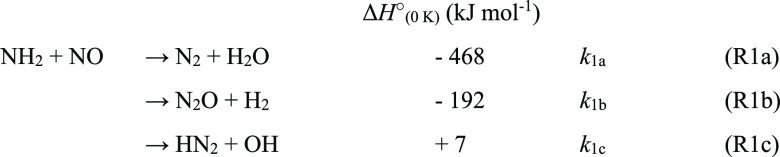



Product channel R1a, which involves breaking all three chemical bonds in the reactants and forming three new chemical bonds in the products, is chain terminating, converting two radical species (NH_2_ + NO) into two closed shell species (N_2_ + H_2_O). Conversely, the product channel R1c is chain propagating, as it produces two new radical species (HN_2_ + OH). As such, the branching ratio (BR) between channels R1a and R1c, α = *k*_1c_/*k*_1_ (where *k*_1_ is the sum of all product channels, *k*_1_ = *k*_1a_ + *k*_1b_ + *k*_1c_), plays an important role in determining the effectiveness of the NH_3_ reducing agent in the DeNOx cycle. Both experiment and theory suggest channel R1b to be insignificant even at very high temperatures; this is despite the N_2_O + H_2_ channel itself being 192 kJ mol^–1^ exothermic, and due to several high activation barriers on the potential energy surface (PES) leading to this channel that lie above the NH_2_ + NO entrance channel.^16,29,31,34^ After the initial association of NH_2_ with NO, the reaction is believed to take place as a single elementary step without collisions, as evidenced by numerous theoretical studies which indicate that the lifetimes of the intermediates are shorter than the time between collisions at temperatures and pressures of interest and by experimental studies which indicate that the rate coefficient is independent of pressure between a few Torr and an atmosphere.^16,20,25^ The HN_2_ radical formed via channel R1c has been shown both experimentally^37^ and theoretically^35,38,39^ to have a short lifetime (<0.5 μs) and promptly dissociates to H + N_2_ by tunneling through a small potential energy barrier.

Looking at the available experimental data on the reaction, measurements have been made over a wide range of temperatures, from 200 K up to around 2800 K. These measurements can be broadly divided into two groups: the lower temperature range up to around 1000 K, in which experiments are carried out in reactors or flow cells and the reaction is monitored directly,^6,8,10,13,15,16,18,21,24,25^ and the higher temperature range around 1000 K and above, in which experiments are carried out in shock tubes or flames and in which the data requires interpretation using high-temperature kinetic modeling.^7,9,11,12,14,17,19,20,22,23^ Although there are some discrepancies in the rate coefficients determined in some earlier works, more recent works from both groups are typically in good agreement and show that the reaction has a negative temperature dependence, with the rate coefficient falling from around 3 × 10^–11^ to 1 × 10^–12^ cm^3^ molecule^–1^ s^–1^ between 200 and 2500 K. The BR between channels R1a and R1c has also been examined in a large number of experimental studies;^6,11–16,18,20–23,25^ again despite a number of discrepancies in some of the earlier works, it is now clear from a number of more recent studies that the BR α increases from around 0.1 at room temperature to around 0.8 at 2500 K. Of the numerous theoretical studies investigating the reaction, only a limited number have attempted to predict this BR.^10,28,31,34^ These studies again indicate that the BR α increases with increasing temperature.

Both NH_2_ and NO have been detected in a variety of astrochemical environments, with nitrogen hydrides and nitrogen oxides both thought to play important roles as nitrogen reservoirs and to potentially participate in the formation of more complex species. NH_2_ radicals have been detected in diffuse molecular clouds^40^ and in high-density star-forming and protostellar regions.^41^ Models are often unable to explain both the absolute and relative abundances of the NHx hydrides,^41^ and gas phase reactions with species such as NO may help to resolve these discrepancies. For example, a gas-phase only model of a diffuse molecular gas cloud underpredicts the observed abundances of both NH and NH_2_ by factors of between 10 and 100, while including processes on dust surfaces increases the abundances of these two species, also results in a failure to match the high NH/NH_3_ ratio observed.^41^ NO, after its first detection toward SgrB2 in 1978,^42^ has been detected in dark clouds, star-forming regions, protostellar envelopes, and nuclei of starburst galaxies (e.g.^43–47^). Despite the possible importance of the reaction between NH_2_ and NO in affecting the partitioning of nitrogen between different reservoirs (being a reaction that converts a nitrogen hydride and a nitrogen oxide into N_2_), the absence of the reaction in the Kinetic Database for Astrochemistry^48^ suggests the reaction is likely missing in many astrochemical models. The reaction is, however, included in the UMIST Database for Astrochemistry (UdFA), with the recommended rate coefficient listed as appropriate at temperatures from 210 K upward.

In this paper, we present measurements of rate coefficients for the reaction between NH_2_ and NO in the temperature range 24–106 K, collected using a pulsed laser-photolysis laser-induced fluorescence (PLP-LIF) technique coupled with a Laval nozzle to achieve the low temperatures relevant to the ISM. We also perform a theoretical investigation into the reaction, recreating the ab initio results of an earlier study to produce a PES and combining this with reaction rate theory using the MESMER software package^49^ to calculate both rate coefficients and BRs over a wide temperature range. These data were then incorporated into a single-point gas-phase model for cold to warm molecular cloud conditions, and the new results were compared with those predicted by current models.^50^

## 2 Methodology

### 2.1 Experimental Study

The use of a Laval nozzle expansion coupled with a PLP-LIF technique has been employed by this group to study the kinetics of a range of neutral–neutral reactions at very low temperatures, including reactions of OH with unsaturated hydrocarbons^51^ and oxygenated volatile organic compounds,^52–55^^1^CH_2_ with atmospheric gases and hydrocarbons,^56,57^ both CH^58^ and CN^59^ with CH_2_O, and NH_2_ with CH_2_O.^60^ In the current study, we employ the same technique to study the low-temperature kinetics of the reaction of NH_2_ with NO. As the experimental apparatus employed in the current and previous studies has been discussed in detail elsewhere,^51–54^ only a brief overview is given here.

The reagent and bath gases were combined in a mixing manifold using calibrated mass flow controllers (MFCs; MKS Instruments) prior to being entered into a 2 L gas ballast tank. The NO reagent was introduced as a pure gas, while the NH_3_ precursor was introduced as either a pure gas or as a dilute mixture of between 10 and 50% in Ar. Following the gas ballast, the reaction mixture was introduced to a 1 cm^3^ stainless-steel reservoir via two pulsed solenoid valves (Parker 9 series) fired at a repetition rate of either 5 or 10 Hz with a pulse duration of around 10 ms. Each pulse of gas underwent a controlled expansion through a convergent-divergent-shaped Laval nozzle into a low-pressure stainless-steel cylindrical chamber (∼775 mm length by 240 mm diameter), resulting in a thermalized low-temperature gas flow. A range of nozzles were employed during the experiments to achieve flow temperatures of between 24 and 106 K. The temperature and density profile of the flows were characterized by impact pressure measurements using a Pitot tube, and the temperature of several of the jets was confirmed by rotationally resolved LIF spectroscopy.^56,58^ The pressure in the vacuum chamber, as measured by two calibrated capacitance manometers (Leybold Ceravac CTR100N 0–10 Torr and Laybold Ceravac CTR90 0–1000 Torr), was typically in the range of 0.2–1.5 Torr depending on the nozzle and flow conditions employed and controlled by adjusting the pumping speed on the screw pump (Edwards GXS160).

**Figure 1:**
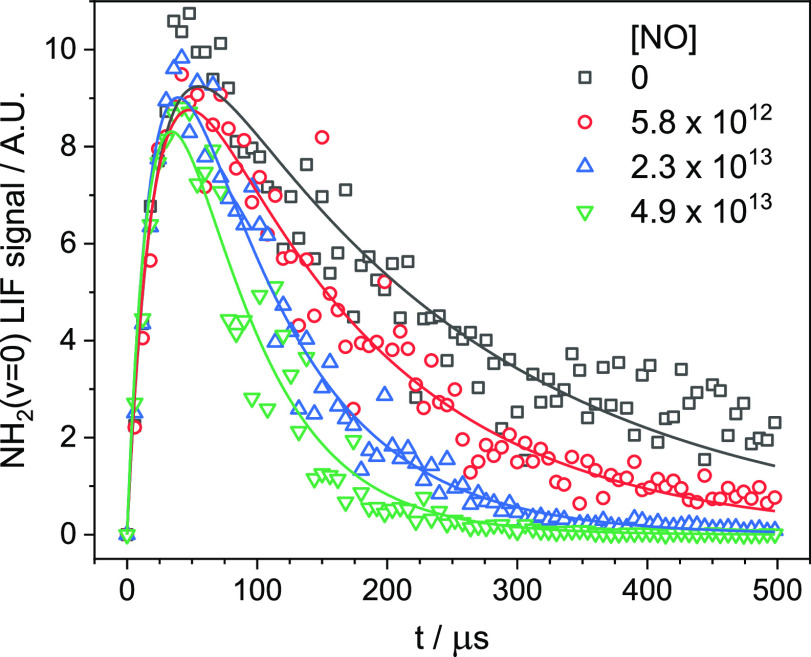
NH_2_ (ν = 0) LIF signal following PLP of NH_3_ at various [NO], given in units of molecules cm^–3^. Solid lines are fits of eq E1 to the data. *T* = 28.2 K, total Ar density = 3.20 × 10^16^ molecules cm^–3^.

NH_2_ radicals were generated from the PLP of NH_3_ at 213 nm (Reaction R2) by the fifth harmonic of a Nd/YAG laser (Quantel Q-smart 850), with a typical pulse energy of ∼10 mJ. The photolysis laser was introduced colinearly with the axis of the expanded gas flow to produce a uniform radical density. NH_2_ radicals were observed by time-resolved LIF spectroscopy, probing the *A*^2^A_1_ (0,9,0) ← *X*^2^B_1_ (0,0,0) transition near 597.6 nm^61,62^ using the output of a Nd/YAG-pumped dye laser (a Quantel Q-smart 850 pumping a Sirah Cobra-Stretch). The probe laser was introduced perpendicularly to the photolysis laser beam, crossing the gas flow at the furthest distance from the exit of the nozzle before the flow broke up due to turbulence. This point was determined from impact pressure measurements as the point at which the density of the flow begins to sharply decline and was typically between 10 and 30 cm, depending on the nozzle and bath gas used. The nonresonant fluorescence at ∼620 nm was collected via a series of lenses through an optical filter (Semrock Brightline interference filter, λ_max_ = 620 nm, fwhm = 14 nm) and observed by a temporally gated channel photomultiplier (CPM; PerkinElmer C1952P), mounted at 90^°^ to both laser beams. The signal from the CPM was recorded using a digital oscilloscope (LeCroy Waverunner LT264) and sent to a computer using a custom LabView program. The temporal evolution of the LIF signal was recorded by varying the time delay between photolysis and probe lasers. A typical time-resolved LIF profile (Figure 1) consisted of between 110 and 165 delay steps and resulted from the average of between 6 and 15 individual delay scans.
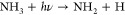
R2

Photolysis of NH_3_ produces both ground and vibrationally excited NH_2_,^62,63^ and, as a consequence of this, the profiles of NH_2_ (*v* = 0) exhibit a growth resulting from the relaxation of vibrationally excited NH_2_ (see Figure 1). In experiments in which only the NH_3_ precursor and the bath gas were present, this growth was on the order of 20,000 s^–1^, indicating that it takes ∼170 μs for 95% of the vibrationally excited NH_2_ to be relaxed down to ν = 0. This posed a problem for kinetic measurements of NH_2_ in our system, as the time scale for kinetic experiments was restricted by the length of the uniform supersonic flow, with dynamic times in our system ranging from ∼200 to 500 μs depending on the nozzle and bath gas used. Therefore, the addition of a species effective in the vibrational relaxation of NH_2_ was required in order to measure accurate rate coefficients for the removal of NH_2_ (ν = 0). Yamasaki et al.^64^ have shown that CF_4_ efficiently accelerates vibrational relaxation of NH_2_. We have found that CH_4_ is also efficient at relaxing vibrationally excited NH_2_. By adding up to 3% CH_4_ to our flows, we achieved a growth of the NH_2_ (ν = 0) signal on the order of between 50,000 and 100,000 s^–1^ (depending on the particular flow), allowing us to effectively monitor the removal of NH_2_ (ν = 0) within the time scales of our experiments. To ensure adding up to 3% CH_4_ to our flows did not significantly affect the temperature and density profiles of our low-temperature gas flows, several flows were characterized by impact pressure measurements both with and without 3% CH_4_ present; the temperatures and densities determined for the flows with CH_4_ present were shown to be within 5% of those determined without CH_4_ present.

### 2.2 Materials

N_2_ (99.9995%, BOC), Ar (99.9995%, BOC), NH_3_ (99.98%, BOC), and NO (99.5%, BOC).

### 2.3 Theoretical Calculations

All electronic structure calculations were carried out using the Gaussian 09 suite of programs.^65^ Geometric structures of the stationary points [reactants, products, and intermediates, including adducts and transition states (TSs)] given in Diau and Smith^29^ were optimized at the B3LYP/6-311G(d,p) level^66–69^ to obtain vibrational frequencies, rotational constants, and electronic and zero point energies (ZPEs). TSs were found to have only one imaginary vibrational frequency, while for the reactants, products, and intermediates, all of the vibrational frequencies were positive. Diau and Smith^29^ also demonstrated that the TSs were connected to their designated intermediates by IRC calculations. ZPEs obtained from the harmonic frequencies were corrected with a scaling factor of 0.967 for B3LYP/6-311G(d,p).^70^ Rate theory calculations to predict rate coefficients and BRs were performed using the master equation solver for multi-energy well reaction (MESMER) program.^49^ Further details of the parameters used in the MESMER program, such as the energy transfer parameters Δ*E*_down_, are given in the MESMER input file attached as part of the Supporting Information. However, as discussed below, as the reaction between NH_2_ + NO is effectively pressure-independent over pressure ranges of interest, the choice of the magnitude of the energy transfer parameters does not play a role in the rate coefficients and BRs calculated.

## 3 Results

**Figure 2:**
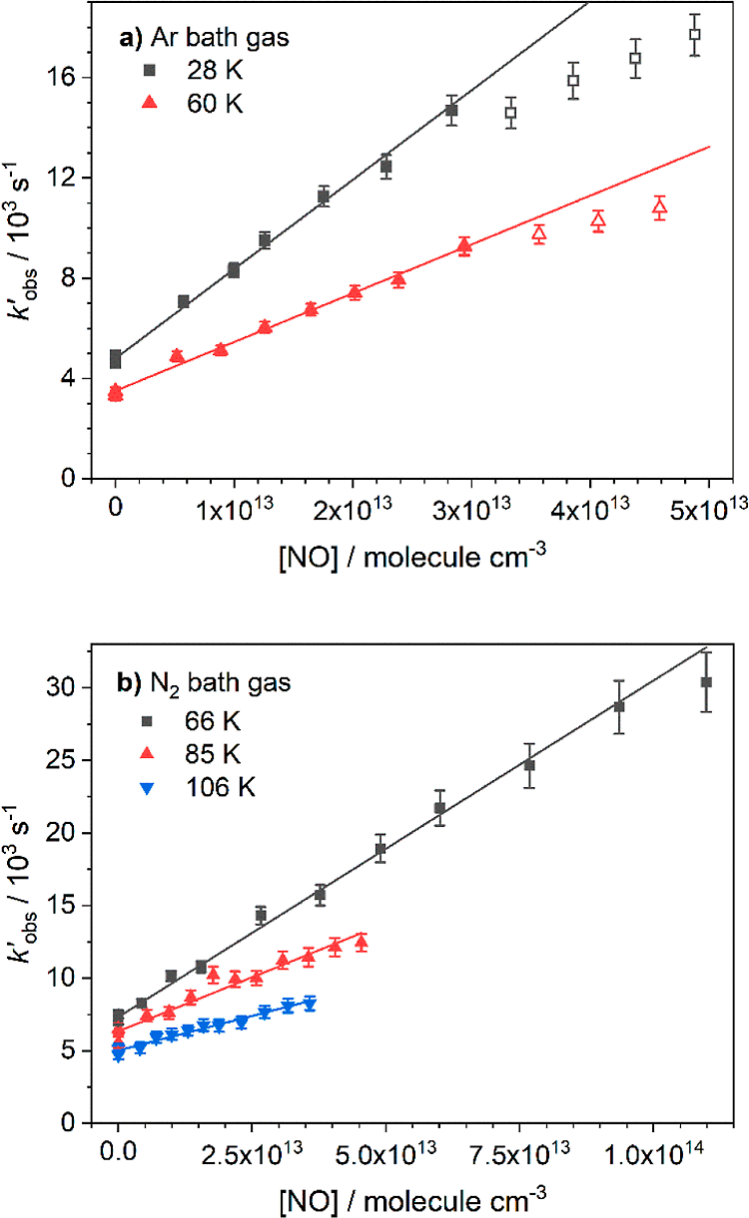
Bimolecular plots of *k*_obs_′ vs [NO] collected at various temperatures, using either (a) Ar or (b) N_2_ as a bath gas. The solid symbols indicate the linear range from which the bimolecular rate coefficient, *k*_1_, is determined, while the open symbols demonstrate curvature caused by the presence of NO dimers and are excluded from the linear fit. No curvature was observed when N_2_ was used as a bath gas.

Typical NH_2_ LIF profiles produced following the photolysis of NH_3_ can be seen in Figure 1. As can be seen from these profiles, there is an initial growth of the NH_2_ signal with no instant signal observed. As discussed above, this growth in the NH_2_ (ν = 0) signal is due to relaxation of vibrationally excited NH_2_ produced following photolysis of NH_3_. Relaxation of vibrationally excited NH_2_ occurs via a cascade, such that ν = *x* → ν = *x* – 1 → ν = *x* – 2 →···→ ν = 0. As such, the growth of the NH_2_ (ν = 0) signal is not strictly a single exponential growth. However, as higher vibrational levels are relaxed faster than lower levels (for a particular collider),^63^ the relaxation of NH_2_ (ν = 1) to NH_2_ (ν = 0) is the rate-limiting step, and as such, the growth of the NH_2_ (ν = 0) signal can be treated as a single-exponential growth (R3)
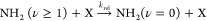
R3where X can be CH_4_, the bath gas, NH_3_, and the coreagent R under investigation. In practice, relaxation by the bath gas (either Ar or N_2_) is slow,^71,72^ necessitating the addition of CH_4_ to our flows to promote efficient vibrational relaxation. Reaction R3, together with the reaction for the removal of NH_2_ (ν = 0)
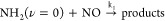
R1and as experiments were carried out under pseudo-first-order conditions (i.e., [NH_2_] ≪ [NO] and [CH_4_]), the temporal evolution of the NH_2_ (ν = 0) LIF signal is given by
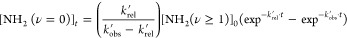
E1and
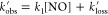
E2where *k*_rel_′ and *k*_obs_′ are the pseudo-first-order rate coefficients for the reactions producing and removing NH_2_ (ν = 0), [NH_2_ (ν ≥ 1)]_0_ is the initial amount of NH_2_ (ν ≥ 1) produced following photolysis of NH_3_ that is subsequently relaxed down to NH_2_ (ν = 0), and *k*_loss_′ is the total rate coefficient for other loss processes of NH_2_ (ν = 0), such as diffusion out of the probe laser beam volume. Equation E1 was fitted to the NH_2_ (ν = 0) LIF profiles, and the parameters *k*_rel_′, *k*_obs_′, and [NH_2_ (ν ≥ 1)]_0_ were extracted. When fitting these NH_2_ profiles, the *k*_rel_′ values were fit globally; that is, for a certain experimental run in which the total pressure, temperature, precursor concentration, and [CH_4_] were kept constant, all the NH_2_ profiles were fit using a single *k*_rel_′ value that was allowed to float. As can be seen from Figure 1, the NH_2_ traces are satisfactorily fit using a biexponential function, indicating the validity of treating the growth of the NH_2_ (ν = 0) signal as a single process. Plotting *k*_obs_′ vs [NO] should then yield a straight line as given by eq E2, with a gradient equal to the bimolecular rate constant, *k*_1_, and intercept *k*_loss_′. Examples of such bimolecular plots can be seen in Figure 2. As can be seen from Figure 2, in some of our bimolecular plots, we observe a curvature in the *k*_obs_′ values at high [NO]. We attribute this curvature to the presence of NO dimers, which are formed in our low-temperature flows at high [NO]. The [NO] at which NO dimers appear in a particular flow will depend on the bath gas used and the temperature and density of the flow, with dimers more likely to form at lower temperatures, higher densities, and with heavier bath gases that act better as third bodies. Indeed, we only observe this curvature in our lowest temperature flows that use Ar as a bath gas, while no curvature was observed in our higher temperature flows using N_2_ as a bath gas. The negative curvature at higher [NO] implies that NO dimers do not remove NH_2_ fast enough to counterbalance the loss of NO monomers (i.e., that the NO dimer removes NH_2_ less than twice as fast as the NO monomer). In bimolecular plots in which curvature was observed, only the linear part of the plot was used to determine *k*_1_ (e.g., solid points in Figure 2), while the other points were excluded (e.g., open points in Figure 2).

An alternative explanation for the curvature that we observe in some of our bimolecular plots is that it is the result of our data fitting procedure rather than due to the presence of NO dimers. At higher [NO], when the kinetic decays become faster, it may be that the NH_2_ (ν ≥ 0) relaxation is not complete, resulting in NH_2_ (ν = 0) decays that may appear slower than expected. To determine if this is the case, we have also fit single exponentials to the NH_2_ traces at long times (i.e., from times at which all of the NH_2_ (ν ≥ 1) should have been relaxed). Rate coefficients, *k*_1_, determined fitting single exponential decays to the data were typically within 10% of those determined when using biexponential fits, with many values in much better agreement. In addition, we typically still observe a curving over of the bimolecular plot at higher [NO] when using data fitted with a single exponential as well as when fitted with a biexponential. As such, it is likely that this curving over is in fact a result of NO dimerization rather than an artifact of our data fitting procedure. This NO dimerization has not been reported in other low-temperature gas expansion experiments using a Laval nozzle,^73–75^ although the characteristics of each low-temperature expansion do differ. NO dimers do, however, form readily in a number of low-temperature molecular beam experiments (e.g.^76,77^), although it should be noted that the temperatures in these experiments are likely significantly lower in our low-temperature flows. The NO dimer itself also has a relatively large binding of ∼14 kJ mol^–1^ for such a small molecule.^78^ In either case, whether this curving over is the result of NO dimers or a data analysis artifact, it is the result of a deviation from the true rate coefficient, warranting the exclusion of these points from the bimolecular plot in our determination of *k*_1_.

**Figure 3:**
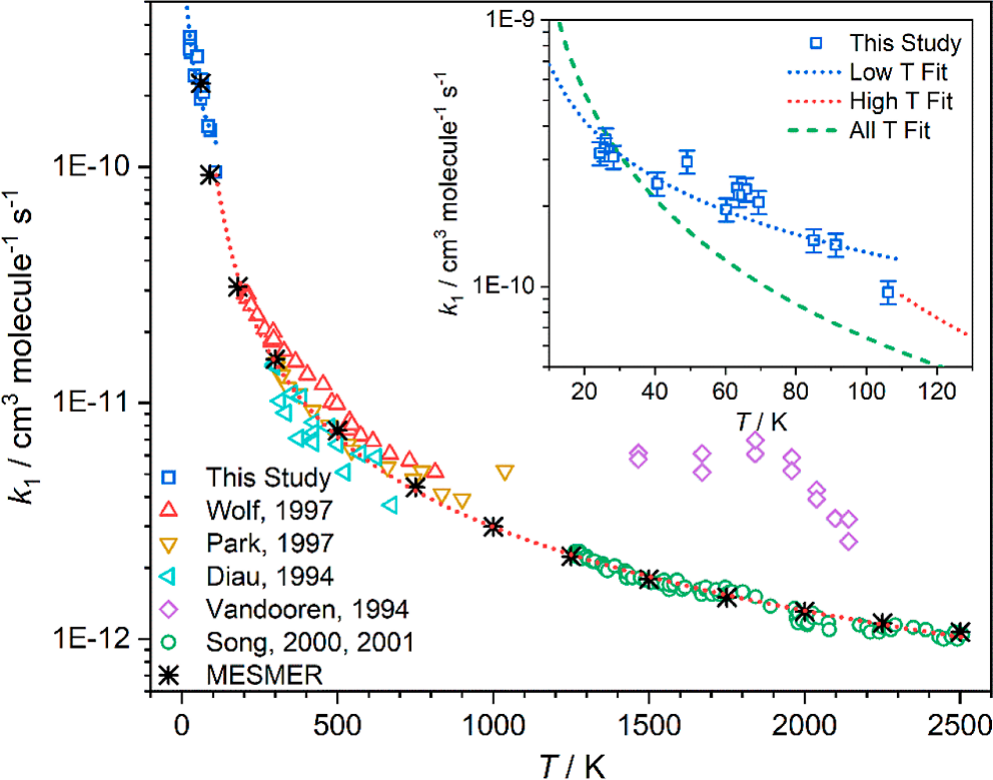
Temperature dependence of the rate coefficient for NH_2_ + NO (Reaction R1). Blue squares: this study. Red upward triangles: Wolf, 1997.^25^ Yellow downward triangles: Park, 1997.^16^ Turquoise left pointing triangles: Diau, 1994.^10^ Purple diamonds: Vandooren, 1994.^22^ Green circles: Song, 2000^19^ and 2001.^20^ Black stars: MESMER rate-theory predictions; see Section 4 for details. The inset is zoomed in over the temperature range investigated in this study. Blue and red dotted lines are the parametrized fits to the low- and high-temperature data. The green dashed line is a parametrized fit to all the data (see text). Errors have been left off the main plot for clarity.

The bimolecular rate coefficients for the reaction of NH_2_ with NO (*k*_1_, Reaction R1) determined in this study are presented in Table 1 and compared with some of the more recent literature data in Figure 3. The errors reported are the 1 σ confidence intervals of linear least-squares fits of the bimolecular plots and do not include systematic errors. No change in the rate coefficient was observed as the total pressure of the flows varied by around a factor of 4, which is consistent with previous studies of the system.^10,25^

**Table 1:** Rate Coefficients for the Reaction of NH_2_ + NO and Relevant Experimental Conditions

∼*T*/K	*T*^a^/K	bath gas	*N*_total_^a^/10^16^ molecules cm^–^^3^	dynamic time/μs	[CH_4_]/10^15^ molecules cm^–^^3^	[NO] range^b^/10^13^ molecules cm^–^^3^	*k*_1_(*T*)^c^/10^–^^10^ cm^3^ molecule^–^^1^ s^–^^1^
26	28 ± 2	Ar	3.2 ± 0.4	∼500	0.63	2.8 (4.9)	3.6 ± 0.1
	26 ± 2	Ar	4.2 ± 0.5	∼500	0.83	2.4 (3.8)	3.8 ± 0.2
	26 ± 2	Ar	6.2 ± 0.5	∼500	1.3	2.2 (6.2)	3.4 ± 0.5
	24 ± 2	Ar	7.7 ± 0.6	∼500	1.6	2.6 (8.5)	3.4 ± 0.5
	41 ± 3	Ar	8.6 ± 0.8	∼250	1.3	3.3 (5.9)	2.5 ± 0.2
	49 ± 4	Ar	6.3 ± 0.8	∼225	1.2	3.2 (3.7)	2.9 ± 0.2
	60 ± 8	Ar	7.9 ± 1.5	∼270	1.3	2.9 (4.6)	2.0 ± 0.1
66	69 ± 2	N_2_	2.2 ± 0.2	∼400	0.43	10.3	2.1 ± 0.1
	66 ± 2	N_2_	3.7 ± 0.2	∼400	0.71	11.0	2.3 ± 0.1
	63 ± 2	N_2_	4.9 ± 0.4	∼400	0.97	9.1	2.4 ± 0.1
	64 ± 2	N_2_	6.5 ± 0.4	∼360	1.3	11.5	2.2 ± 0.1
	85 ± 2	N_2_	7.5 ± 0.4	∼180	1.2	4.5	1.5 ± 0.1
	91 ± 6	N_2_	4.9 ± 0.9	∼280	0.90	2.5	1.4 ± 0.1
	106 ± 9	N_2_	6.7 ± 1.5	∼200	1.2	3.6	0.95 ± 0.05

a Uncertainties in each value of *T* and *N*_total_ are ±1 σ (the standard deviation) of the measured temperature and density along the axis of the Laval expansion.

b Gives the [NO] range from 0 to the stated value that was used in determining *k*_1_, while the value in brackets gives the full range of [NO] used in the experiment (if different).

c Uncertainties for each value of *k*(*T*) reported at the 1 σ level for the linear least-squares fitting of the pseudo-first-order rate coefficients as a function of [NO].

There have been many previous studies investigating the removal of NH_2_ with NO over temperatures ranging from 200 to 2500 K, employing a range of experimental techniques, with the results presented here being the first low-temperature measurements below 200 K. Figure 3 presents some of the more recent temperature-dependent rate coefficients given in the literature. The highest temperature data above ∼1200 K are from two shock tube studies carried out by Song et al.,^19,20^ in which the NH_2_ is detected using frequency modulation absorption spectroscopy, and from a study by Vandooren et al.,^22^ in which either the NO consumption or N_2_ production in a low-pressure ammonia-nitric oxide flame was monitored by mass spectrometry, with the rate coefficients extracted following high-temperature kinetic modeling of the data. The midtemperature range data between ∼200 and 1100 K come from three studies by Wolf et al.,^25^ Diau et al.,^10^ and Park and Lin,^16^ who, following PLP of NH_3_ in the presence of NO, monitor either the removal of NH_2_ or the growth of H_2_O using LIF, cavity ring down spectroscopy, and mass spectrometry, respectively.

As can be seen from Figure 3, our low-temperature data follow the general trend of the higher-temperature data, with the rate of removal of NH_2_ by NO increasing with decreasing temperature. Parameterizing the data over the entire temperature range did not give a satisfactory fit to the low-temperature data obtained in this study (see dashed green line in the inset of Figure 3). In particular, an all-temperature parametrization does not take into account the apparent slowdown in the increase in rate coefficient with decreasing temperature, and extrapolating such a fit down to 10 K yields a rate coefficient that is around 4 times faster than that measured at 25 K and that is approaching the theoretical classical capture theory collision limit (estimated to be 10^–9^ cm^3^ molecule^–1^ s^–1^ at 10 K, by comparison with a similarly sized system).^59^ Instead, both the low-temperature data below 110 K and the high-temperature data above 200 K were parametrized separately (eqs E3 and E4) using a modified Arrhenius expression, giving the blue and red dotted lines shown in Figure 3, respectively. This resulted in a significantly better fit of the low-temperature data provided in this study and takes account of the apparent slowdown in the increase of the rate coefficient. Additionally, extrapolation of the low-temperature parametrized fit yields a rate coefficient at 10 K around twice as fast as that determined at 25 K, and that is well below the theoretical collision limit. All experimental data points were given a 10% error when parametrizing the data to ensure no one data point at a particular temperature was overly weighted. With the exception of the Vandooren et al. data, which report considerably larger rate coefficients than the other studies over a similar temperature range, the majority of the experimental data points lie within 20% of the parametrized rate coefficients, with most deviating by only a few percent. The low- and high-temperature parametrized rate coefficients are given by (errors are 1 σ):
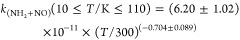
E3
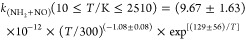
E4

## 4 Discussion

### 4.1 Theoretical Calculations

**Figure 4:**
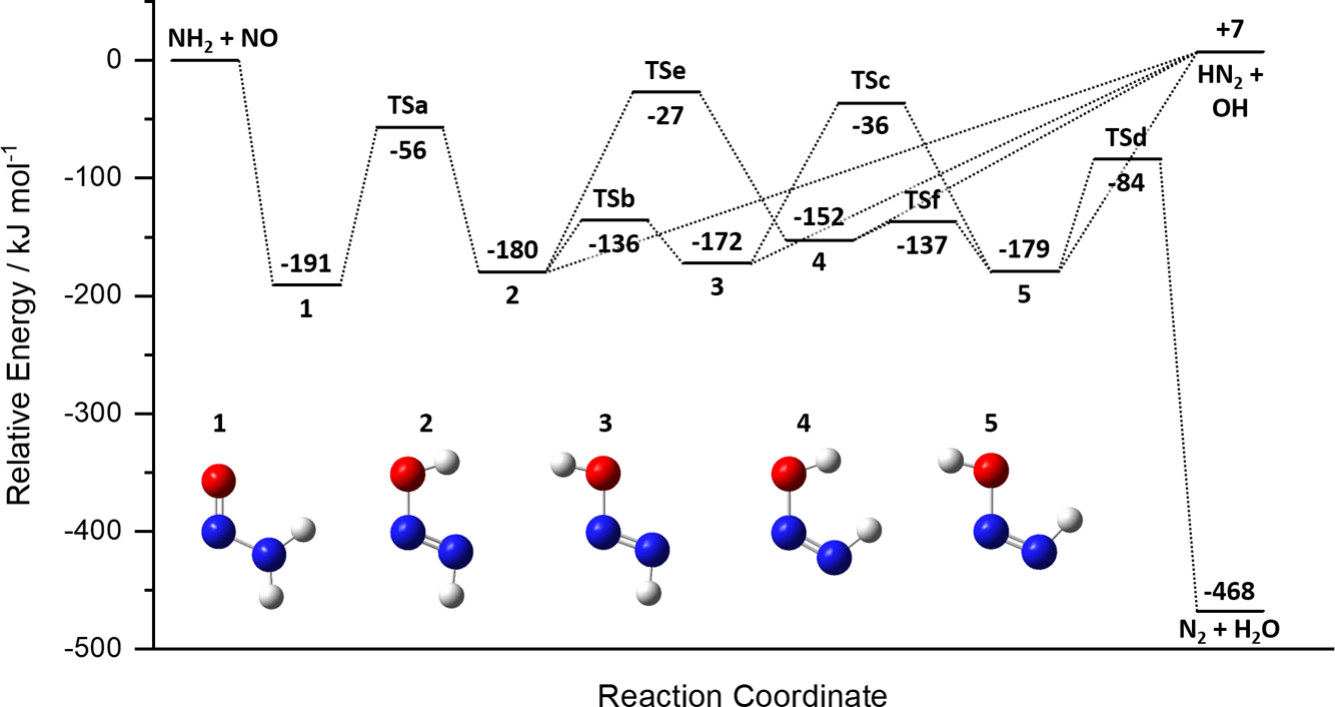
PES for NH_2_ + NO, determined at the B3LYP/6-311G(d,p) level of theory. The N_2_O + H_2_ product channel (R1b) and the TSs and stationary points leading to it have been excluded (see the text for details).

For the reaction between NH_2_ and NO, there have been several theoretical investigations, with a number of PESs calculated at various levels of theory.^10,24–36^ For this study, we have taken the structures given in Diau and Smith^29^ and optimized them at the B3LYP/6-311G(d,p) level in order to obtain vibrational frequencies, rotational constants, and electronic and ZPEs, details of which can be found in the MESMER input file, which is included in the Supporting Information. The calculated energies and vibrational frequencies are in excellent agreement with those reported by Diau and Smith^29^ using the same level of theory. Table S1 compares our calculated energies to some of the more recent calculated energies in the literature.^29,31^ As can be seen from Table S1, although B3LYP/6-311G(d,p) could be considered a relatively low level of theory by modern computational standards, the energies calculated at this level of theory are in reasonable agreement with those from higher-level calculations. Comparing the energies of the adducts 1–5 and the TSs a–f (see Figure 4), the B3LYP energies are typically within 15 kJ mol^–1^ of those calculated at the G2M(CC1) level of theory^79^ by Diau and Smith^29^ and in even better agreement with those calculated at the CCSD(T)/aug-cc-pVTZ level of theory by Fang et al.,^31^ being within 9 kJ mol^–1^. Comparing the energies of the key three stationary points (TSs c and e, and the HN_2_ + OH exothermicity, see discussion below), we typically see even better agreement between the B3LYP and higher-level calculations, and indeed, when fitting these parameters to the experimental data (see below), only minor adjustments to these energies are required. As such, we have chosen not to refine the B3LYP energies by carrying out higher-level electronic energy calculations.

The NH_2_ + NO PES is complex, with many deep potential wells and large barriers and three possible product channels

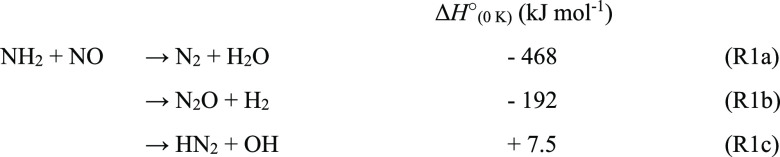



A schematic of the full PES for the reaction can be seen in the Supporting Information (Figure S2). As discussed above, the TSs leading to the N_2_O + H_2_ product channel (R1b) all lie above the NH_2_ + NO entrance channel. As such, both experiment and theory suggest channel R1b to be a very minor channel, even at temperatures as high as 2500 K. Indeed, preliminary MEMSER calculations carried out using the full PES indicated that channel R1b accounts for less than 0.2% of the total rate coefficient at 2500 K. As such, all further MESMER calculations were carried out using the reduced surface shown in Figure 4, in which the stationary points leading to channel R1b are excluded. As can be seen from Figure 4, the reaction is initiated by the barrierless addition of NH_2_ to the NO to form the adduct H_2_NNO (structure 1, Figure 4). This adduct undergoes a H atom shift followed by a series of cis–trans isomerizations, giving rise to four distinct HNNOH isomers. All four of these isomers (structures 2–5 in Figure 4) may undergo an endothermic, barrierless bond fission to form the products HN_2_ + OH, while only one of the isomers (no. 5) is configured correctly to form the N_2_ + H_2_O products via a four-membered ring TS.

### 4.2 Rate Theory Calculations Using MESMER

**Figure 5:**
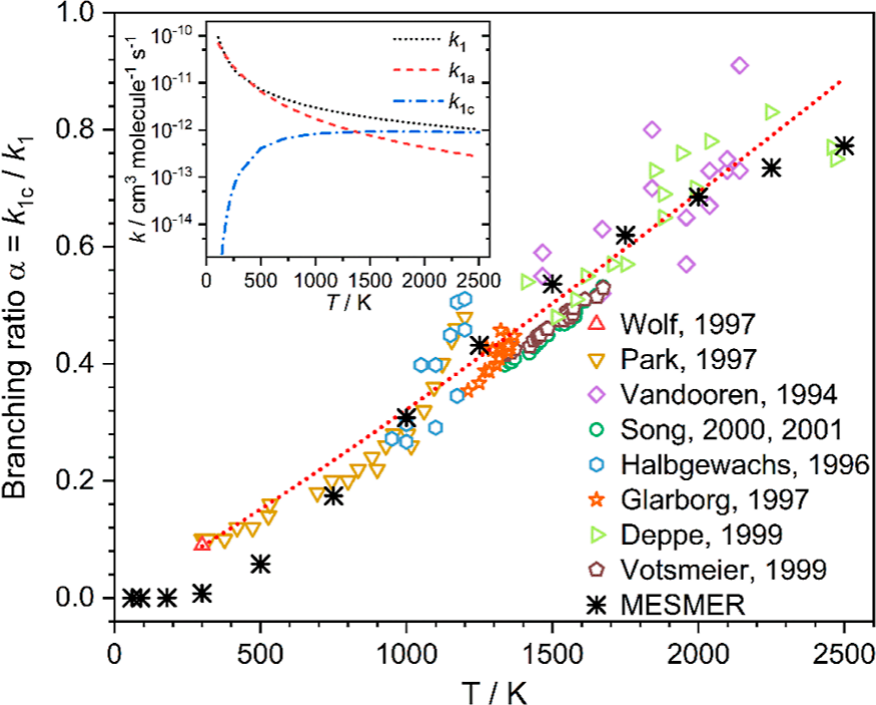
Experimentally determined BR α = *k*_1c_/*k*_1_ as a function of temperature. Experimental error bars have not been included for clarity but are quoted below where given. Red upward triangles: Wolf, 1997, error given as ±0.03.^25^ Yellow downward triangles: Park, 1997, errors not given.^16^ Purple diamonds: Vandooren, 1994, errors not given.^22^ Green circles: Song, 2000^19^ and 2001, errors not given.^20^ Blue hexagons: Halbgewachs 1996, average quoted error ±0.01.^12^ Orange stars: Glarborg 1997, average quoted error ±0.03.^11^ Light green left facing triangles: Deppe, errors not given, 1997.^9^ Brown pentagons: Votsmeier, 1999, estimated average error ±0.05.^23^ Black stars: MESMER predictions. The red dotted line is a parametrized fit to literature data (eq E5). The inset shows the temperature dependence of the rate coefficients over the temperature range *T* = 110–2510 K for; black dotted line: total NH_2_ + NO removal rate coefficient (*k*_1_). Red dashed line: product channel was N_2_ + H_2_O (*k*_1a_, eq E6). Blue dot dash line: product channel HN_2_ + OH (*k*_1c_, eq E7).

Using the program MESMER, we can fit the experimental data by allowing various parameters used in the rate theory calculation or features of the PES itself to be adjusted. In this manner, we are able to improve the agreement between the experimental and calculated rate coefficients and BRs. Rather than fitting all of the available experimental data, in order to reduce the computational time required when carrying out the rate theory calculations, fitting was carried out using only a limited number of rate coefficient and product BR values, as given by the parametrized fits (eqs E3–E5) to the experimental data (see Figures 3 and 5). The temperatures and values used in the fitting, together with the MESMER predicted values, are given in Table 2. The parameters that were adjusted, together with the values obtained from the fitting, are given in Table 3. Initially, each parameter was fitted independently to assess its effect on the predicted rate coefficients/BRs, before a final fitting was carried out in which all the parameters were floated together. When parametrizing the experimental BR data from the literature (see Figure 5), each data point was given a ± 0.1 error to ensure no one data point at a particular temperature was overly weighted, giving (errors are 1 σ)
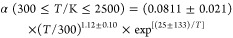
E5The key parameter adjusted when fitting the experimental rate coefficients were the inverse Laplace transform (ILT) parameters for the initial association reaction of NH_2_ with NO, which take the form of a modified Arrhenius function *A*(*T*/300)^*n*^ (with the activation energy being set to zero for the barrierless process). The final fitting returned values of *A*_1_ = 2.02 × 10^–11^ cm^3^ molecule^–1^ s^–1^ and *n*_1_ = −0.977 for the initial association reaction of NH_2_ with NO, giving a satisfactory fit to rate coefficients over almost the entire temperature range (60–2500 K, see Figure 3), with the predicted rate coefficients obtained using these *A* and *n* values typically being within 10% of the rates given by the parametrized fits (eqs E3 and E4; see Table 2). Due to the NH_2_ + NO PES containing many deep wells, MESMER was unable to predict or fit rate coefficients and BRs below 60 K.

**Table 2:** Comparison of the Rate Coefficient for the NH_2_ + NO Reaction and BR α = *k*_1c_/*k*_1_ Calculated Using MESMER with the Values Given by the Parametrized Fits to the Experimental Data (eqs E3–E5)

*T*/K	*k*_1_/cm^3^ molecule^–^^1^ s^–^^1^			BR α for HN_2_ + OH product channel (R1c)		
	parameterized fits^a^	MESMER	% difference (%)	parameterized fit	MESMER	difference
60	1.92 × 10^–^^10^	2.26 × 10^–^^10^	17.7		0.000	
90	1.45 × 10^–^^10^	9.27 × 10^–^^11^	36.1		0.000	
180	3.44 × 10^–^^11^	3.11 × 10^–^^11^	9.6		0.000	
300	1.49 × 10^–^^11^	1.53 × 10^–^^11^	2.7	0.075	0.008	0.067
500	7.19 × 10^–^^12^	7.64 × 10^–^^12^	6.3	0.137	0.058	0.079
750	4.26 × 10^–^^12^	4.41 × 10^–^^12^	3.5	0.220	0.175	0.045
1000	2.99 × 10^–^^12^	3.00 × 10^–^^12^	0.3	0.306	0.308	0.002
1250	2.28 × 10^–^^12^	2.24 × 10^–^^12^	1.8	0.395	0.432	0.037
1500	1.84 × 10^–^^12^	1.79 × 10^–^^12^	2.7	0.487	0.536	0.049
1750	1.54 × 10^–^^12^	1.50 × 10^–^^12^	2.6	0.580	0.620	0.04
2000	1.32 × 10^–^^12^	1.31 × 10^–^^12^	0.8	0.675	0.685	0.01
2250	1.15 × 10^–^^12^	1.17 × 10^–^^12^	1.7	0.772	0.735	0.037
2500	1.02 × 10^–^^12^	1.07 × 10^–^^12^	4.9	0.870	0.773	0.097

a Rate coefficients calculated using both the low (<110 K, E3) and high (>110 K, E4) temperature parametrized fits.

**Table 3:** Parameters Adjusted during the Fitting to the Experimental Data in the Rate Theory Calculations together with the Values Obtained from the Fitting (Errors are 1 σ). See Text for Details

parameter	B3LYP^b^	GM2^c^	CCSD(T)^d^	fitting^e^
*A*_1_/cm^3^ molecule^–^^1^ s^–^^1^	(2.12 ± 0.11) × 10^–^^11^	(1.81 ± 0.07) × 10^–^^11^	(2.95 ± 0.23) × 10^–^^11^	(2.02 ± 0.19) × 10^–^^11^
*n*_1_	–0.960 ± 0.040	–1.090 ± 0.030	–0.774 ± 0.059	–0.977 ± 0.044
*A*_2_/cm^3^ molecule^–^^1^ s^–^^1^	(8.12 ± 1.41) × 10^–^^12^	(3.97 ± 0.90) × 10^–^^11^	(1.25 ± 0.26) × 10^–^^11^	(1.30 ± 0.69) × 10^–^^11^
*n*_2_	–0.101 ± 0.034	–0.075 ± 0.031	–0.198 ± 0.149	–0.079 ± 0.069
HN_2_ + OH ZPE/kJ mol–^1^	7.5 (fixed)	15.1 (fixed)	10.5 (fixed)	9.6 ± 3.5
TSe ZPE/kJ mol^–^^1^	–26.7 (fixed)	–33.9 (fixed)	–18.4 (fixed)	–30.8 ± 8.4
TSc ZPE^a^/kJ mol^–^^1^	–36.2 (fixed)	–45.6 (fixed)	–32.6 (fixed)	–40.3 ± 8.4 (linked^f^)
χ^2^ (degrees of freedom)^a^	1.127 (19)	0.983 (19)	1.070 (19)	1.293 (17)

a Values given are χ^2^/degrees of freedom (experimental points minus parameters, given in brackets).

b Values obtained using B3LYP/6-311G(d,p) energies.

c Values obtained using G2M(CC1) energies.^29^

d Values obtained using CCSD(T)/aug-cc-pVTZ energies.^31^

e Values obtained using B3LYP/6-311G(d,p) energies and allowing the energies of the key stationary points to float.

f TSs e and c were not floated independently. Instead, TSe was allowed to float while TSc was derived from TSe, meaning both would move up and down in step with each other.

We have identified several parameters that are likely to affect the BR α for the HN_2_ + OH product channel predicted by MESMER. Key among these are the reverse ILT parameters used for the rate coefficients of the barrierless dissociation reactions of the four HNNOH isomers (nos. 2−5 in Figure 4). These parameters, *A*_2_ and *n*_2_, again take the form of a modified Arrhenius function, *A*(*T*/300)^*n*^. Although the *A* and *n* parameters for the dissociation reaction of each distinct HNNOH isomer could be varied individually, we have used the same *A* and *n* values for all four reactions due to the similarity in the dissociation reactions, as has been done in previous theoretical investigations.^25,34^ Indeed, in the more recent theoretical investigation of this reaction by Fang et al.,^31^ in which they do treat the dissociation of the cis and trans isomers separately, they indicate that the dissociation rate coefficients are typically within 30% of one another, indicating that this simplification should not represent too large an error. Fitting of the experimental data returned values of *A*_2_ = 1.30 × 10^–11^ cm^3^ molecule^–1^ s^–1^ and *n*_2_ = −0.079, indicating little temperature dependence on the dissociation rate. In addition to the reverse ILT parameters, two other parameters have been shown to be important factors in determining the BR α; the HN_2_ + OH endothermicity and the heights of the barriers linking the cis–trans isomers (TSe and TSc).^31,34^ When adjusting these parameters, we treat the cis–trans isomer barriers as a pair, allowing the energies of both to move up or down in tandem while maintaining the absolute difference in energy between the two TSs, as attempting to float both TSs independently resulted in significantly larger errors in all of the fitted parameters, suggesting that the results were not defined. These two parameters (the HN_2_ + OH endothermicity and the TSe/TSc barrier heights) in some ways compensate each other. For example, by lowering the heights of the TSe/TSc pair, we effectively promote the cis–trans isomerization processes, leading to adduct 5, which promptly dissociates to N_2_ + H_2_O, resulting in a decrease in α. However, this may be countered by decreasing the HN_2_ + OH product endothermicity, which in effect promotes this channel, resulting in an increase in α. How these two parameters are adjusted has also been shown to affect the shape of the BR vs T plot (Figure 5) by affecting how α increases with temperature. The best fit to the experimental data required increasing the HN_2_ + OH exothermicity by 2.1 kJ mol^–1^ (from 7.5 to 9.6 kJ mol^–1^) and decreasing the heights of the TSe/TSc pair by 4.1 kJ mol^–1^ (from −26.7 to −30.8 kJ mol^–1^ and from −36.2 to −40.3 kJ mol^–1^ for TSe and TSc, respectively). These are much smaller changes than those made by Fang et al.,^31^ in which they move the ZPEs of the HN_2_ + OH channel and the TSe/TSc pair, as calculated at the CCSD(T)/aug-cc-pVTZ level of theory, down by 7 and 17 kJ mol^–1^, respectively. This in effect moves their values from being around 5 kJ mol^–1^ higher than our calculated values to around 6 kJ mol^–1^ lower than our values following fitting. It should be noted that at the level of theory employed in this study, we would expect errors of around 10 kJ mol^–1^ in our calculated energies.

In addition to floating all of the parameters discussed above, we have also carried out some additional runs in which we only allow the ILT parameters to float while fixing the energies of the stationary points on the PES to either the B3LYP/6-311G(d,p) values determined in this study, the G2M(CC1) values determined by Diau and Smith^29^ or the CCSD(T)/aug-cc-pVTZ values determined by Fang et al.^31^ in order to compare the surfaces from the three levels of theory. Table 3 gives the values of the fitted parameters together with the goodness of each fit (the χ^2^ values). As can be seen from Table 3, we were able to obtain reasonable fits to the experimental data by fitting the ILT parameters only when using energies from all three levels of theory, with only a slight improvement to the fit when using the higher levels of theory. This is unsurprising considering how similar the energies of the stationary points are at each level of theory (Table S1). What is slightly surprising is that we actually see a small decrease in the quality of the fit when allowing all of the parameters to float when using the B3LYP energies; we put this down to the fact that there are now more degrees of freedom and that the parameters we chose to float (the HN_2_ + OH endothermicity and the TSe/TSc barrier heights) only required minor adjustments to achieve a good fit to the experimental data.

BRs predicted by MESMER using the values obtained from the fitting of the experimental data are given in Table 2 and shown in Figure 5. As can be seen from Figure 5, there is reasonable agreement between the experimental and calculated BRs over the entire temperature range of available experimental data (300–2500 K), with the predicted BRs lying within ±0.1 of the parametrized experimental values (eq 5; see Table 2). The largest discrepancies between the experimental and theoretical BRs appear at both the high and low ends of the temperature range, with the predicted BRs showing a more pronounced sinusoidal shape than is reflected by the experimental data. Fang et al.^31^ also noted the difficulty in calculating a BR that is in good agreement with the literature data at room temperature, suggesting the possibility of experimental error in the room-temperature measurements or other possible shortcomings in the theoretical model, such as understanding the dissociation rates of the different isomers or the treatment of the density of states of some of the TSs, which is used in the calculation of the rate coefficient. However, despite these shortcomings, we are able to predict temperature-dependent BRs that are largely consistent with experiment. Extending the temperature range over which we predict BRs down to 60 K, we can see that as the temperature drops to 180 K and below, the BR α has reached zero, indicating that the endothermic HN_2_ + OH product channel (R1c) is unviable at these low temperatures; conversely, the BR for the low energy channel producing N_2_ + H_2_O (R1a) reaches 1 at 180 K and below. As such, the low-temperature parametrized fit given in Section 3 can be taken as having only one product channel, R1a, producing N_2_ + H_2_O.

In order to produce rate coefficient expressions for the two product channels open at higher temperatures, we have applied the BRs predicted by MESMER to the rate coefficients given by the high-temperature parametrized fit in Section 3 (eq E4) to give channel-specific rate coefficients. These channel-specific rate coefficients can be parametrized as (see inset Figure 5; errors are 1 σ level of a least-squares fit to the data)
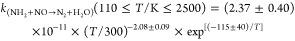
E6
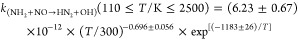
E7

These rate coefficients and BRs predicted by MESMER are, in effect, the zero pressure rate coefficients and BRs that are applicable to the interstellar medium (ISM). This was confirmed by the fact that rate coefficients and BRs calculated between 60 and 2500 K were shown to be pressure independent over the range 1 × 10^12^ to 1 × 10^20^ molecules cm^–3^; only at pressures of 1 × 10^21^ molecules cm^–3^ and above was any pressure dependence observed.

### 4.3 Astrochemical Implications

The rate coefficients and product channels for the NH_2_ + NO reaction were added to a chemical network used for modeling interstellar and circumstellar material.^80^ The gas-phase chemistry in this network is based on the Rate12 release of the UMIST Database for Astrochemistry.^50^ A series of single-point gas-phase only chemical models were run, covering the temperature (10–30 K) and density (10^4^–10^6^ molecules cm^–3^) ranges typical of cold to warm molecular clouds. Fully shielded conditions were assumed (i.e., a high visual extinction), and a cosmic-ray ionization rate of 1.3 × 10^–17^ s^–1^ was used. Initial abundances are taken from Table 3 in McElroy et al.^50^ The models were run until a time of 10^8^ years to steady state. A comparison of the results obtained with and without the updated rate coefficients showed no major changes in the abundances of the reactants (NH_2_ and NO) nor products (H_2_O and N_2_), with maximal differences on the order of ∼1%. At a temperature of 10 K, Rate12 adopts a recommended rate coefficient of 1.49 × 10^–12^ cm^3^ molecule^–1^ s^–1^, which is around 450 times lower than the updated value from this work (6.70 × 10^–10^ cm^3^ molecule^–1^ s^–1^). The fact that no species involved in the reaction mechanism exhibits major changes in their abundances using this faster rate coefficient shows that this reaction is not a major loss mechanism for NH_2_ nor NO and, correspondingly, is not a major production mechanism for H_2_O nor N_2_ under molecular cloud conditions. Inspection of the dominant reactions at 10 K and at steady state showed that loss of NH_2_ is dominated by reaction with O to form OH or HNO, whereas loss of NO is dominated by reaction with atomic N to form N_2_.

## 5 Conclusions

The reaction between NH_2_ and NO has been studied experimentally over the temperature range 24–106 K using a pulsed laser-photolysis laser-induced fluorescence (PLP-LIF) technique coupled with a Laval nozzle expansion in order to reach the low temperatures of the ISM. The reaction has been shown to have a negative temperature dependence, in agreement with higher temperature literature data over the temperature range 200–2500 K. Ab initio calculations of the PES of the NH_2_ + NO system were carried out, with the structures of the stationary points on the PES given in Diau and Smith^29^ optimized in order to obtain vibrational frequencies, rotational constants, and electronic and ZPEs. Using this surface, rate theory calculations were carried out using the MESMER software package. Experimentally determined rate coefficients and BRs have been used to fit the calculated PES. Good agreement between the calculated and experimental rate coefficients was obtained over the entire temperature range by simply adjusting the ILT parameters for the initial association reaction of the NH_2_ with NO. Fitting to the experimental BRs from the literature required only minor adjustments to the calculated PES to be made, moving the HN_2_ + OH exothermicity down by 2.1 kJ mol^–1^, and the TSe and TSc pair (which control the cis–trans isomerization reactions of the HNNOH adducts) down by 4.1 kJ mol^–1^. In this manner, we were able to predict BRs that are largely consistent with experimentally determined literature (within ±0.1) values over a wide range of temperatures. The calculated BRs show a more pronounced sinusoidal shape than is reflected in the experimental data; as such, the largest discrepancies occur at both the high (2500 K) and low (300 K) temperature ends of the temperature range. Extending the temperature range over which we predict BRs down to 60 K, we see that at 180 K and below, the HN_2_ + OH endothermic product channel is no longer open, and the reaction proceeds along the low-temperature channel, forming N_2_ + H_2_O. Despite the relatively fast reaction rate measured at low temperatures in this work (∼10^–10^ cm^3^ s^–1^), inclusion of the new rates in astrochemical models showed that this reaction is not an important loss mechanism for NH_2_ nor NO under molecular cloud conditions; however, the impact of the newly measured rate constant should be tested in other low-temperature astrophysical environments in which radical species are abundant, e.g., photon-dominated regions or the outer winds of AGB stars.

## Supporting Information Available

Comparison of relative energies of stationary points on the NH_2_ + NO PES from this and other recent literature studies; full PES for NH_2_ + NO; and energies calculated at the B3LYP/6-311G(d,p) level of theory (PDF)

## References

[ref1] Glarborg P.; Damjohansen K.; Miller J. A.; Kee R. J.; Coltrin M. E. Modeling the thermal DENOx process in flow reactors. Surface effects and Nitrous Oxide formation. Int. J. Chem. Kinet. 1994, 26, 421–436. 10.1002/kin.550260405.

[ref2] Lyon R. K. The NH3-NO-O2 reaction. Int. J. Chem. Kinet. 1976, 8, 315–318. 10.1002/kin.550080213.

[ref3] Miller J. A.; Bowman C. T. Mechanism and modeling of nitrogen chemistry in combustion. Prog. Energy Combust. Sci. 1989, 15, 287–338. 10.1016/0360-1285(89)90017-8.

[ref4] Miller J. A.; Branch M. C.; Kee R. J. A chemical kinetic model for the selective reduction of nitric oxide by ammonia. Combust. Flame 1981, 43, 81–98. 10.1016/0010-2180(81)90008-0.

[ref5] Miller J. A.; Glarborg P. Modeling the Thermal De-NOx Process: Closing in on a final solution. Int. J. Chem. Kinet. 1999, 31, 757–765. 10.1002/(SICI)1097-4601(1999)31:11<757::AID-JCK1>3.0.CO;2-V.

[ref6] Atakan B.; Jacobs A.; Wahl M.; Weller R.; Wolfrum J. Kinetic measurements and product branching ratio for the reaction NH_2_+NO at 294–1027 K. Chem. Phys. Lett. 1989, 155, 609–613. 10.1016/0009-2614(89)87482-2.

[ref7] Brown M. J.; Smith D. B. Aspects of nitrogen flame chemistry revealed by burning velocity modelling. Symp. (Int.) Combust., [Proc.] 1994, 25, 1011–1018. 10.1016/S0082-0784(06)80738-1.

[ref8] Bulatov V. P.; Ioffe A. A.; Lozovsky V. A.; Sarkisov O. M. On the reaction of the NH_2_ radical with NO at 295–620 K. Chem. Phys. Lett. 1989, 161, 141–146. 10.1016/0009-2614(89)85046-8.

[ref9] Deppe J.; Friedrichs G.; Romming H. J.; Gg Wagner H. A kinetic study of the reaction of NH_2_ with NO in the temperature range 1400–2800 K. Phys. Chem. Chem. Phys. 1999, 1, 427–435. 10.1039/a808390h.

[ref10] Diau E. W.; Yu T.; Wagner M. A. G.; Lin M. C. Kinetics of the NH_2_ + NO Reaction: Effects of Temperature on the Total Rate Constant and the OH/H_2_O Branching Ratio. J. Phys. Chem. 1994, 98, 4034–4042. 10.1021/j100066a022.

[ref11] Glarborg P.; Kristensen P. G.; DamJohansen K.; Miller J. A. Branching fraction of the NH_2_+NO reaction between 1210 and 1370 K. J. Phys. Chem. A 1997, 101, 3741–3745. 10.1021/jp970264g.

[ref12] Halbgewachs M. J.; Diau E. W. G.; Mebel A. M.; Lin M. C.; Melius C. F. Thermal reduction of NO by NH_3_: Kinetic modeling of the NH_2_+NO product branching ratio. Symp. (Int.) Combust., [Proc.] 1996, 26, 2109–2115. 10.1016/S0082-0784(96)80035-X.

[ref13] Hall J. L.; Zeitz D.; Stephens J. W.; Kasper J. V. V.; Glass G. P.; Curl R. F.; Tittel F. K. Studies of the NH_2_ + NO reaction by infrared kinetic spectroscopy. J. Phys. Chem. 1986, 90, 2501–2505. 10.1021/j100402a047.

[ref14] Kimballlinne M. A.; Hanson R. K. Combustion-driven flow reactor studies of thermal DeNOx reaction kinetics. Combust. Flame 1986, 64, 337–351. 10.1016/0010-2180(86)90150-1.

[ref15] Park J.; Lin M. C. Direct determination of product branching for the NH_2_+NO reaction at temperatures between 302 and 1060 K. J. Phys. Chem. 1996, 100, 3317–3319. 10.1021/jp9533741.

[ref16] Park J.; Lin M. C. Laser-Initiated NO Reduction by NH_3_: Total Rate Constant and Product Branching Ratio Measurements for the NH_2_ + NO Reaction. J. Phys. Chem. A 1997, 101, 5–13. 10.1021/jp961568q.

[ref17] Roose T. R.; Hanson R. K.; Kruger C. H. A shock tube study of the decomposition of no in the presence of NH_3_. Symp. (Int.) Combust., [Proc.] 1981, 18, 853–862. 10.1016/S0082-0784(81)80089-6.

[ref18] Silver J. A.; Kolb C. E. Kinetic measurements for the reaction of amidogen + nitric oxide over the temperature range 294-1215 K. J. Phys. Chem. 1982, 86, 3240–3246. 10.1021/j100213a033.

[ref19] Song S.; Hanson R. K.; Bowman C. T.; Golden D. M. Shock tube determination of the overall rate of NH_2_ + NO → products at high temperatures. Proc. Combust. Inst. 2000, 28, 2403–2409. 10.1016/S0082-0784(00)80653-0.

[ref20] Song S.; Hanson R. K.; Bowman C. T.; Golden D. M. Shock tube determination of the overall rate of NH_2_ + NO → products in the thermal De-NOx temperature window. Int. J. Chem. Kinet. 2001, 33, 715–721. 10.1002/kin.1068.

[ref21] Stephens J. W.; Morter C. L.; Farhat S. K.; Glass G. P.; Curl R. F. Branching ratio of the reaction NH_2_ + NO at elevated temperatures. J. Phys. Chem. 1993, 97, 8944–8951. 10.1021/j100137a019.

[ref22] Vandooren J.; Bian J.; Van Tiggelen P. J. Comparison of experimental and calculated structures of an ammonia-nitric oxide flame. Importance of the NH_2_ + NO reaction. Combust. Flame 1994, 98, 402–410. 10.1016/0010-2180(94)90178-3.

[ref23] Votsmeier M.; Song S.; Hanson R. K.; Bowman C. T. A shock tube study of the product branching ratio for the reaction NH_2_ + NO using frequency-modulation detection of NH_2_. J. Phys. Chem. A 1999, 103, 1566–1571. 10.1021/jp983613v.

[ref24] Wolf M.; Yang D. L.; Durant J. L. Kinetic-studies of NH_x_ radical reactions. J. Photochem. Photobiol., A 1994, 80, 85–93. 10.1016/1010-6030(93)01037-3.

[ref25] Wolf M.; Yang D. L.; Durant J. L. A Comprehensive Study of the Reaction NH_2_ + NO → Products: Reaction Rate Coefficients, Product Branching Fractions, and ab Initio Calculations. J. Phys. Chem. A 1997, 101, 6243–6251. 10.1021/jp9713165.

[ref26] Abou-Rachid H.; Pouchan C.; Chaillet M. Ab initio CI study of the reaction between NH_2_ and NO. Chem. Phys. 1984, 90, 243–255. 10.1016/0301-0104(84)85323-9.

[ref27] Casewit C. J.; Goddard W. A. Energetics and mechanisms for reactions involving nitrosamide, hydroxydiazenes, and diimide N-oxides. J. Am. Chem. Soc. 1982, 104, 3280–3287. 10.1021/ja00376a005.

[ref28] Diau E. W. G.; Smith S. C. Temperature dependence of rate coefficients and branching ratios for the NH_2_ + NO reaction via microcanonical variational transition state theory. J. Phys. Chem. 1996, 100, 12349–12354. 10.1021/jp9602991.

[ref29] Diau E. W. G.; Smith S. C. Theoretical investigation of the potential energy surface for the NH_2_ + NO reaction via density functional theory and ab initio molecular electronic structure theory. J. Chem. Phys. 1997, 106, 9236–9251. 10.1063/1.474025.

[ref30] Duan X.; Page M. Theoretical characterization of structures and vibrational frequencies for intermediates and transition states in the reaction of NH_2_ with NO. J. Mol. Struct.: THEOCHEM 1995, 333, 233–242. 10.1016/0166-1280(94)03953-I.

[ref31] Fang D. C.; Harding L. B.; Klippenstein S. J.; Miller J. A. A direct transition state theory based analysis of the branching in NH_2_ + NO. Faraday Discuss. 2001, 119, 207–222. 10.1039/b102235k. 11877992

[ref32] Harrison J. A.; Maclagan R.; Whyte A. R. Structures, energies, and vibrational frequencies of intermediates and transition-states in the reaction of NH_2_ and NO. J. Phys. Chem. 1987, 91, 6683–6686. 10.1021/j100311a025.

[ref33] Melius C. F.; Binkley J. S. Energetics of the reaction pathways for NH_2_ + NO → products and NH + NO → products. Symp. (Int.) Combust., [Proc.] 1985, 20, 575–583. 10.1016/S0082-0784(85)80546-4.

[ref34] Miller J. A.; Klippenstein S. J. Theoretical Considerations in the NH_2_ + NO Reaction. J. Phys. Chem. A 2000, 104, 2061–2069. 10.1021/jp992836y.

[ref35] Phillips L. F. A priori rate-constant for the reaction NH_2_ + NO → N_2_ + H_2_O. Chem. Phys. Lett. 1987, 135, 269–274. 10.1016/0009-2614(87)85154-0.

[ref36] Walch S. P. Theoretical characterization of the reaction NH_2_ + NO → products. J. Chem. Phys. 1993, 99, 5295–5300. 10.1063/1.465972.

[ref37] Selgren S. F.; McLoughlin P. W.; Gellene G. I. Observation of dissociative and radiative states of N_2_H by neutralized ion-beam techniques. J. Chem. Phys. 1989, 90, 1624–1629. 10.1063/1.456054.

[ref38] Koizumi H.; Schatz G. C.; Walch S. P. A coupled channel study of HN_2_ unimolecular decay based on a global ab initio potential surface. J. Chem. Phys. 1991, 95, 4130–4135. 10.1063/1.460768.

[ref39] Walch S. P.; Duchovic R. J.; Rohlfing C. M. Theoretical characterization of the minimum energy path for hydrogen-atom addition to N_2_ - implications for the unimolecular lifetime of HN_2_. J. Chem. Phys. 1989, 90, 3230–3240. 10.1063/1.455875.

[ref40] Vandishoeck E. F.; Jansen D. J.; Schilke P.; Phillips T. G. Detection of the interstellar NH_2_ radical. Astrophys. J. 1993, 416, L83–L86. 10.1086/187076.

[ref41] Persson C. M.; De Luca M.; Mookerjea B.; Olofsson A. O. H.; Black J. H.; Gerin M.; Herbst E.; Bell T. A.; Coutens A.; Godard B.; et al. Nitrogen hydrides in interstellar gas II. Analysis of Herschel/HIFI observations towards W49N and G10.6–0.4 (W31C). Astron. Astrophys. 2012, 543, 34 10.1051/0004-6361/201118686.

[ref42] Liszt H. S.; Turner B. E. Microwave detection of interstellar NO. Astrophys. J., Lett. 1978, 224, L73–L76. 10.1086/182762.

[ref43] Codella C.; Viti S.; Lefloch B.; Holdship J.; Bachiller R.; Bianchi E.; Ceccarelli C.; Favre C.; Jiménez-Serra I.; Podio L.; et al. Nitrogen oxide in protostellar envelopes and shocks: the ASAI survey. Mon. Not. R. Astron. Soc. 2018, 474, 5694–5703. 10.1093/mnras/stx3196.

[ref44] Halfen D. T.; Apponi A. J.; Ziurys L. M. Evaluating the N/O Chemical Network: The Distribution of N_2_O and NO in the Sagittarius B2 Complex. Astrophys. J. 2001, 561, 244–253. 10.1086/322770.

[ref45] Martin S.; Mauersberger R.; Martin-Pintado J.; Garcia-Burillo S.; Henkel C. First detections of extragalactic SO_2_, NS and NO. Astron. Astrophys. 2003, 411, L465–L468. 10.1051/0004-6361:20031442.

[ref46] McGonagle D.; Irvine W. M.; Minh Y. C.; Ziurys L. M. Detection of Nitric Oxide in the Dark Cloud L134N. Astrophys. J. 1990, 359, 121 10.1086/169040. 11538685

[ref47] Ziurys L. M.; McGonagle D.; Minh Y.; Irvine W. M. Nitric Oxide in Star-forming Regions: Further Evidence for Interstellar N-O Bonds. Astrophys. J. 1991, 373, 535 10.1086/170072. 11538086

[ref48] Wakelam V.; Herbst E.; Loison J. C.; Smith I. W. M.; Chandrasekaran V.; Pavone B.; Adams N. G.; Bacchus-Montabonel M. C.; Bergeat A.; Beroff K.; et al. A Kinetic Database for Astrochemistry (KIDA). Astrophys. J., Suppl. Ser. 2012, 199, 21 10.1088/0067-0049/199/1/21.

[ref49] Glowacki D. R.; Liang C.-H.; Morley C.; Pilling M. J.; Robertson S. H. MESMER: An Open-Source Master Equation Solver for Multi-Energy Well Reactions. J. Phys. Chem. A 2012, 116, 9545–9560. 10.1021/jp3051033. 22905697

[ref50] McElroy D.; Walsh C.; Markwick A. J.; Cordiner M. A.; Smith K.; Millar T. J. The UMIST database for astrochemistry 2012. Astron. Astrophys. 2013, 550, A36 10.1051/0004-6361/201220465.

[ref51] Taylor S. E.; Goddard A.; Blitz M. A.; Cleary P. A.; Heard D. E. Pulsed Laval nozzle study of the kinetics of OH with unsaturated hydrocarbons at very low temperatures. Phys. Chem. Chem. Phys. 2008, 10, 422–437. 10.1039/B711411G. 18174984

[ref52] Caravan R. L.; Shannon R. J.; Lewis T.; Blitz M. A.; Heard D. E. Measurements of rate coefficients for reactions of OH with ethanol and propan-2-ol at very low temperatures. J. Phys. Chem. A 2015, 119, 7130–7137. 10.1021/jp505790m. 25216323

[ref53] Gomez Martin J. C.; Caravan R. L.; Blitz M. A.; Heard D. E.; Plane J. M. C. Low Temperature Kinetics of the CH_3_OH + OH Reaction. J. Phys. Chem. A 2014, 118, 2693–2701. 10.1021/jp5002995. 24669816 PMC4190665

[ref54] Shannon R. J.; Blitz M. A.; Goddard A.; Heard D. E. Accelerated chemistry in the reaction between the hydroxyl radical and methanol at interstellar temperatures facilitated by tunnelling. Nat. Chem. 2013, 5, 745–749. 10.1038/nchem.1692. 23965675

[ref55] Shannon R. J.; Caravan R. L.; Blitz M. A.; Heard D. E. A combined experimental and theoretical study of reactions between the hydroxyl radical and oxygenated hydrocarbons relevant to astrochemical environments. Phys. Chem. Chem. Phys. 2014, 16, 3466–3478. 10.1039/C3CP54664K. 24407041

[ref56] Douglas K.; Blitz M. A.; Feng W. H.; Heard D. E.; Plane J. M. C.; Slater E.; Willacy K.; Seakins P. W. Low temperature studies of the removal reactions of 1CH2 with particular relevance to the atmosphere of Titan. Icarus 2018, 303, 10–21. 10.1016/j.icarus.2017.12.023.

[ref57] Douglas K. M.; Blitz M. A.; Feng W.; Heard D. E.; Plane J. M. C.; Rashid H.; Seakins P. W. Low temperature studies of the rate coefficients and branching ratios of reactive loss vs quenching for the reactions of 1CH2 with C2H6, C2H4, C2H2. Icarus 2019, 321, 752–766. 10.1016/j.icarus.2018.12.027.

[ref58] West N. A.; Millar T. J.; Van de Sande M.; Rutter E.; Blitz M. A.; Decin L.; Heard D. E. Measurements of Low Temperature Rate Coefficients for the Reaction of CH with CH_2_O and Application to Dark Cloud and AGB Stellar Wind Models. Astrophys. J. 2019, 885, 134 10.3847/1538-4357/ab480e.

[ref59] West N. A.; Li L. H. D.; Millar T. J.; Van de Sande M.; Rutter E.; Blitz M. A.; Lehman J. H.; Decin L.; Heard D. E. Experimental and theoretical study of the low-temperature kinetics of the reaction of CN with CH_2_O and implications for interstellar environments. Phys. Chem. Chem. Phys. 2023, 25, 7719–7733. 10.1039/D2CP05043A. 36876874 PMC10015628

[ref60] Douglas K. M.; Lucas D. I.; Walsh C.; West N. A.; Blitz M. A.; Heard D. E. The Gas-phase Reaction of NH_2_ with Formaldehyde (CH_2_O) is not a Source of Formamide (NH_2_CHO) in Interstellar Environments. Astrophys. J., Lett. 2022, 937, L16 10.3847/2041-8213/ac8cef.

[ref61] Copeland R. A.; Crosley D. R.; Smith G. P. In Laser-induced fluorescence spectroscopy of NCO and NH2 in atmospheric pressure flames. Symposium (International) on Combustion; Elsevier, 1985; pp 1195–1203.

[ref62] Donnelly V. M.; Baronavski A. P.; McDonald J. R. ArF laser photodissociation of NH3 at 193 nm: internal energy distributions in NH2 X2B1 and A2A1, and two-photon generatin of NH A 3Π and b 1Σ+. Chem. Phys. 1979, 43, 271–281. 10.1016/0301-0104(79)85194-0.

[ref63] Yamasaki K.; Watanabe A.; Kakuda T.; Itakura A.; Fukushima H.; Endo M.; Maruyama C.; Tokue I. Vibrational energy distributions of NH_2_ (X^2^*B*_1_) fragments generated in the photolysis of NH_3_ at 193 nm: Application of kinetic analysis on vibrational cascade. J. Phys. Chem. A 2002, 106, 7728–7735. 10.1021/jp025927v.

[ref64] Yamasaki K.; Watanabe A.; Tanaka A.; Sato M.; Tokue I. Kinetics of the reaction NH_2_ (X^2^*B*_1_, ν_2_ = 0 and 1) + NO. J. Phys. Chem. A 2002, 106, 6563–6569. 10.1021/jp013306g.

[ref65] Frisch M.; Trucks G.; Schlegel H.; Scuseria G.; Robb M.; Cheeseman J.; Scalmani G.; Barone V.; Mennucci B.; Petersson G.; Gaussian 09. Revision A.02; Gaussian, Inc.: Wallingford CT, 2016.

[ref66] Becke A. D. Density-functional thermochemistry. II. The effect of the Perdew-Wang generalized-gradient correlation correction. J. Chem. Phys. 1992, 97, 9173–9177. 10.1063/1.463343.

[ref67] Becke A. D. Density-functional thermochemistry. I. The effect of the exchange-only gradient correction. J. Chem. Phys. 1992, 96, 2155–2160. 10.1063/1.462066.

[ref68] Becke A. D. Density-functional thermochemistry. III. The role of exact exchange. J. Chem. Phys. 1993, 98, 5648–5652. 10.1063/1.464913.

[ref69] Lee C. T.; Yang W. T.; Parr R. G. Development of the Colle-Salvetti correlation-energy formula into a functional of the electron-density. Phys. Rev. B: Condens. Matter Mater. Phys. 1988, 37, 785–789. 10.1103/PhysRevB.37.785. 9944570

[ref70] NIST Computational Chemistry Comparison and Benchmark Database. http://cccbdb.nist.gov/. (accessed April, 2023).

[ref71] Gericke K. H.; Torres L. M.; Guillory W. A. State selected removal of vibrationally excited NH_2_ [*X*^2^B_1_(0,ν2,0)] radicals. J. Chem. Phys. 1984, 80, 6134–6140. 10.1063/1.446714.

[ref72] Xiang T.-X.; Gericke K.-H.; Torres L. M.; Guillory W. A. Vibrational relaxation of NH_2_ |*X*^2^B_1_ (0, *v*2, 0)| radicals. Chem. Phys. 1986, 101, 157–163. 10.1016/0301-0104(86)87031-8.

[ref73] Bocherel P.; Herbert L. B.; Rowe B. R.; Sims I. R.; Smith I. W. M.; Travers D. Ultralow-Temperature Kinetics of CH(X^2^Π) Reactions: Rate Coefficients for Reactions with O_2_ and NO (*T* = 13–708 K), and with NH_3_ (*T* = 23–295 K). J. Phys. Chem. 1996, 100, 3063–3069. 10.1021/jp952628f.

[ref74] Nunez-Reyes D.; Hickson K. M. The reactivity of C(1D) with oxygen bearing molecules NO and O2 at low temperature. Chem. Phys. Lett. 2017, 687, 330–335. 10.1016/j.cplett.2017.09.028.

[ref75] Nunez-Reyes D.; Hickson K. M. A low temperature investigation of the gas-phase N(^2^D) + NO reaction. Towards a viable source of N(^2^D) atoms for kinetic studies in astrochemistry. Phys. Chem. Chem. Phys. 2018, 20, 17442–17447. 10.1039/C8CP02851F. 29911699

[ref76] Tsubouchi M.; de Lange C. A.; Suzuki T. Femtosecond time-resolved charged particle imaging studies of the ultraviolet photodissociation of the NO dimer. J. Chem. Phys. 2003, 119, 11728–11739. 10.1063/1.1624600.

[ref77] Wade E. A.; Cline J. I.; Lorenz K. T.; Hayden C.; Chandler D. W. Direct measurement of the binding energy of the NO dimer. J. Chem. Phys. 2002, 116, 4755–4757. 10.1063/1.1459702.

[ref78] Ivanic J.; Schmidt M. W.; Luke B. High-level theoretical study of the NO dimer and tetramer: Has the tetramer been observed?. J. Chem. Phys. 2012, 137, 214316 10.1063/1.4769226. 23231240 PMC3528699

[ref79] Mebel A. M.; Morokuma K.; Lin M. C. Modification of the Gaussian-2 theoretical-model - the use of coupled-cluster energies, density-functional geometries, and frequencies. J. Chem. Phys. 1995, 103, 7414–7421. 10.1063/1.470313.

[ref80] Walsh C.; Nomura H.; van Dishoeck E. The molecular composition of the planet-forming regions of protoplanetary disks across the luminosity regime. Astron. Astrophys. 2015, 582, A88 10.1051/0004-6361/201526751.

