# Supplementary material for: Experimental and Theoretical Investigation of the Reaction of NH2 with NO at Very Low Temperatures
Source: J Phys Chem A. 2023 Aug 17;127(34):7205–15. doi: 10.1021/acs.jpca.3c03652 (PMC10476206; doi:10.1021/acs.jpca.3c03652)

# Experimental and Theoretical Investigation of the Reaction of $NH_2$ with NO at Very Low Temperatures

Kevin M. Douglas[a], Daniel Lucas[a], Catherine Walsh[b], Mark A. Blitz[a,c], Dwayne E. Heard[a*]

[a]*School of Chemistry, University of Leeds, Leeds, LS2 9JT, UK*

[b]*School of Physics and Astronomy, University of Leeds, Leeds, LS2 9JT, UK*

[c]*National Centre for Atmospheric Science (NCAS), University of Leeds, Leeds, LS2 9JT, UK*

[*]corresponding authors. Email: k.m.douglas@leeds.ac.uk; d.e.heard@leeds.ac.uk

## Supplementary Information

The supplementary information contains:

**Page2. Table S1.** Comparison of relative energies of stationary points on the $NH_2$ + NO potential energy surface from this and other recent literature studies.

**Page3. Figure S2.** Full potential energy surface for $NH_2$ + NO. Energies calculated at the B3LYP/6-311G(d,p) level of theory. The N2O + $H_2$ product channel (R1b) is excluded in the majority of MESMER calculations carried out, see main text for details.

**Table S1.** Comparison of relative energies of stationary points on the $NH_2$ + NO potential energy surface from this and other recent literature studies.

| Species[a] | This work[b] | Diau 97[c] | | | Fang 01[d], CCSD(T) | | | This work adjusted | Fang 01[d] adjusted |
|---|---|---|---|---|---|---|---|---|---|
| | | B3LYP[b] | CCSD(T)[e] | G2M[f] | cc-pvdz[g] | cc-pvtz[h] | aug-cc-pvtz[i] | | |
| 1 | -191.0 | -191.2 | -161.1 | -195.4 | -152.3 | -174.5 | | | |
| 2 | -179.5 | -179.5 | -164.4 | -194.1 | -159.4 | -179.1 | -182.0 | | |
| 3 | -172.2 | -172.4 | -157.7 | -192.5 | -149.8 | -172.8 | -178.7 | | |
| 4 | -152.7 | -152.7 | -136.4 | -168.2 | -136.0 | -154.0 | -155.6 | | |
| 5 | -179.3 | -179.5 | -163.6 | -193.7 | -159.0 | -177.4 | -180.3 | | |
| a | -56.8 | -56.9 | -31.8 | -61.9 | -32.6 | -48.5 | -50.6 | | |
| b | -135.7 | -135.6 | -124.7 | -158.2 | -117.6 | -139.7 | | | |
| **c** | **-36.2** | **-36.0** | **2.5** | **-45.6** | **10.9** | **-21.3** | **-32.6** | **-40.3** | **-49.4** |
| d | -83.8 | -83.7 | -64.4 | -104.2 | -63.6 | -81.2 | -92.5 | | |
| **e** | **-26.7** | **-26.8** | **10.0** | **-33.9** | **18.4** | **-10.5** | **-18.4** | **-30.8** | **-35.1** |
| f | -136.6 | -136.8 | -123.4 | -156.5 | -121.8 | -142.3 | | | |
| $N_2$ + $H_2O$ | -467.6 | -467.8 | -494.1 | -520.1 | -479.9 | -504.2 | -507.9 | | |
| **$HN_2$ + OH** | **7.5** | **7.5** | **15.5** | **15.1** | **18.8** | **13.8** | **10.5** | **9.6** | **3.8** |

[a]All energies are given in kJ mol-1 and include corrected zero-point energies. See Figure 4 for potential energy surface. The energies of the key stationary points floated when fitting to experimental data are in **bold** type.

[b]Calculated at the B3LYP/6-311G(*d,p*) level of theory.

[c]Diau and Smith[1]

[d]Fang*, et al.*[2]

[e]Calculated at the CCSD(T)/6-311G(*d,p*)//B3LYP/6-311G(*d,p*) level of theory.

[f]Calculated at the G2M(CC1)//B3LYP/6-311G(*d,p*) level of theory, see Diau and Smith[1] for details.

[g]Calculated at the CCSD(T)/cc-pvdz level of theory.

[h]Calculated at the CCSD(T)/cc-pvtz level of theory.

[i]Calculated at the CCSD(T)/aug-cc-pvtz//CCSD(T)/cc-pvtz level of theory.

## References


1. Diau, E. W. G.;Smith, S. C. Theoretical investigation of the potential energy surface for the $NH_2$ + NO reaction via density functional theory and ab initio molecular electronic structure theory. *J. Chem. Phys.,*1997, **106,** 9236-9251.
2. Fang, D. C.; Harding, L. B.; Klippenstein, S. J.; Miller, J. A. A direct transition state theory based analysis of the branching in $NH_2$ + NO. *Faraday Discuss.,*2001, **119,** 207-222.

**Figure S2.** Full potential energy surface for $NH_2$ + NO. Energies calculated at the B3LYP/6-311G(d,p) level of theory. The N2O + $H_2$ product channel (R1b) is excluded in the majority of MESMER calculations carried out, see main text for detail.

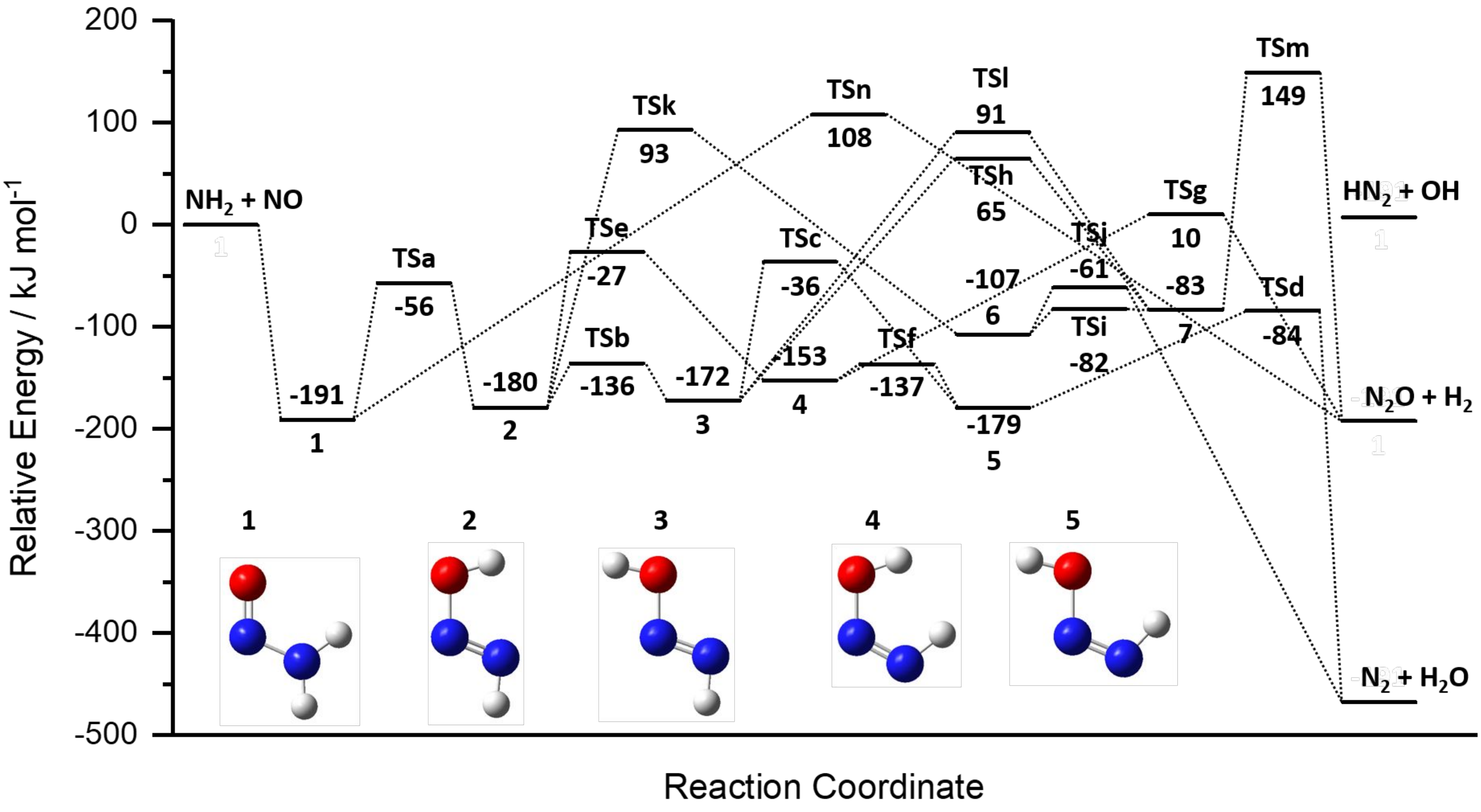

Supplement: Supplementary file 1 — jp3c03652_si_001.pdf [file jp3c03652_si_001.pdf]
